# Spatio-temporal heterogeneity and coupling effect of mining economy, social governance and environmental conservation: Evidence from Guangxi Zhuang Autonomous Region, China

**DOI:** 10.1371/journal.pone.0301585

**Published:** 2024-04-16

**Authors:** Mingkai Liu, Hongyan Zhang, Kaixin Hou, Xiaoju Gong, Changxin Liu

**Affiliations:** 1 School of Economics, Beijing Technology and Business University, Beijing, China; 2 College of Resources and Environmental Economics, Inner Mongolia University of Finance and Economics, Hohhot, Inner Mongolia, China; 3 Institution of Science and Development, China Academy of Sciences, Beijing, China; Khwaja Fareed University of Engineering & Information Technology, PAKISTAN

## Abstract

In order to solve the problem of coordinated development among mining economy, social governance and environmental conservation in global resource-based cities, we choose Guangxi Zhuang Autonomous Region as the research area. The advantage of resource endowment and resource industry was measured by location quotient and input-output method. The panel data related to mining governance from 2010 to 2021 were selected to build the evaluation and coupling analysis model between mining economic, social governance and environmental conservation, and the spatial-temporal heterogeneity and coupling effect of them were analyzed by comprehensive empowerment evaluation, spatial autocorrelation analysis and barrier degree methods. The results show that: (1) Except for the overall upward trend of social governance, the development level of mining economy and environmental conservation are basically stable; (2) The resource-rich areas have obvious mining economic advantages, and the central cities have good social governance capabilities, and the environmental conservation effectiveness is uncertain; (3) The coupling effect between mining economy and social governance is stronger than that between mining economy and environment conservation, and the synergistic coupling effect of the three is relatively random. Finally, we put forward some policy response strategies to Guangxi, and theoretical and practical reference would be provided for resource-based cities around the world.

## 1. Introduction

The core position of resource industry economy has supported the rapid economic growth of resource-based cities in a certain historical period, and also promoted the process of global industrialization [1–3]. However, with the sharp decline of resource reserves and the continuous increase of environmental regulation pressure, resource-based cities are facing the double pressure of resource curse and environmental governance. The resource industry gradually loses its economic advantage, and the region suffers from the risk of *Dutch Disease* [4–6]. Since the 18th National Congress of the Communist Party of China, Chinese leaders have put forward the initiative of "clear waters and lush mountains are gold and silver mountains". The development of resource-based cities must get rid of the problem of resource dependence, and promote the high-quality development of resource-based cities by means of technology-driven and industrial transformation [7]. In addition, the "barrel effect" points out that the factors affecting the overall transformation efficiency are often the key shortcomings, and the situation change of regional coordinated development determines the necessity and urgency of resource-based city transformation [8, 9]. Therefore, it is an important direction to continuously promote the transformation of mining economic structure, reduce the dependence of resource-based cities on the mining industry chain, improve the effect of regional environmental governance, and realize the coupling between mining economy, social governance and environment conservation. It is also a hot scientific issue in the field of economic and social governance of resource-based cities [10–12]. Taking Guangxi Zhuang Autonomous Region of China as an example, this paper studies the spatio-temporal heterogeneity and coupling effects of mining economy, social governance and environmental conservation, which is conducive to providing scientific basis and beneficial suggestions for the high-quality economic and social development of resource-based cities, which is in line with the transformation and development needs of resource-based cities.

The existing researches on mining economy, social security and environmental conservation mainly involve resource industries and resource-based cities, which identify the barriers of interaction between different factors and propose ideas for industrial and regional transformation [13–15]. From the whole life cycle of resource industry, the loss of advantage of resource endowment is the basic factor restricting its sustainable development. Exploration, development, washing, smelting, processing and distribution must be systematically considered to enhance the sustainable development capacity of the resource industry [16–18]. Strengthening resource exploration and development is of great significance to find out the base of resource endowment, but it is also necessary to balance the contradiction between generation effect and supply of resources [19–21]. At the same time, the washing, smelting and processing of resource products are typical extensive production modes, and exploring the two-wheel drive mode of technology enablement and management innovation can help avoid process waste [22, 23]. In addition, the "resource-asset-capital" integration model is considered to be an important way to extend and recycle the resource industry chain. Under the current industrial development situation, the use of capital circulation advantages of resource products can effectively enhance the value of the resource industry chain [24–26]. From the perspective of resource-based city governance, mining economic growth is only a single element of regional coordinated development, especially in the current macro background of tighter resource and environmental policy constraints, the supporting role of the resource industry for high-quality regional economic growth is gradually weakening, and it is necessary to propose classified response strategies according to the actual needs of regional economic and social governance [27, 28]. The process of developing the mining economy in resource-based cities has led to labor employment and technological progress, but it has also caused energy encroachment and environmental damage. Therefore, the simultaneous promotion of mining economy, society and environment is considered to be an effective approach to governance [29–31]. Under the background of complex international situation and frequent sudden events, relying on a single model driven by resource-based industries can no longer adapt to the new development pattern. It is also an important consideration for the transformation of resource-based cities to improve regional economic resilience [32–34].

We holds that the sustainable development of resource-based cities has been unable to rely on resource industry driven, and the constraints of diversified factors lead to differences in the transformation efficiency of resource-based cities. Therefore, this study aims to solve the resource, environment and policy problems in the transformation process of resource-based cities by revealing the interaction between mining economy, society and environmental system. To be specific, following the principle of constructing an evaluation system based on indicators related to the development of the resource industry sector and supplemented by objective environmental indicators, we establishes a systematic evaluation model of mining economy, social governance and environmental conservation; then, the integrated evaluation and analysis methods are used to measure the spatio-temporal evolution trend and coupling effect of factors in the study area, analyze the mechanism of action among the three and identify the coupling constraints of mining economy, social governance and environment; finally, the response strategy of regional economic and social governance is put forward. Although we have carried out a local study on Guangxi, the research results are still universal. As Guangxi is representative of resource-based cities in the world, we hope to put forward suggestions that are consistent with the sustainable development of resource-based cities in other regions of China and even the world. Of course, this study is also an important theoretical research innovation for the development of resource-based cities.

## 2. Methodology

### 2.1 General research design

**Fig 1:**
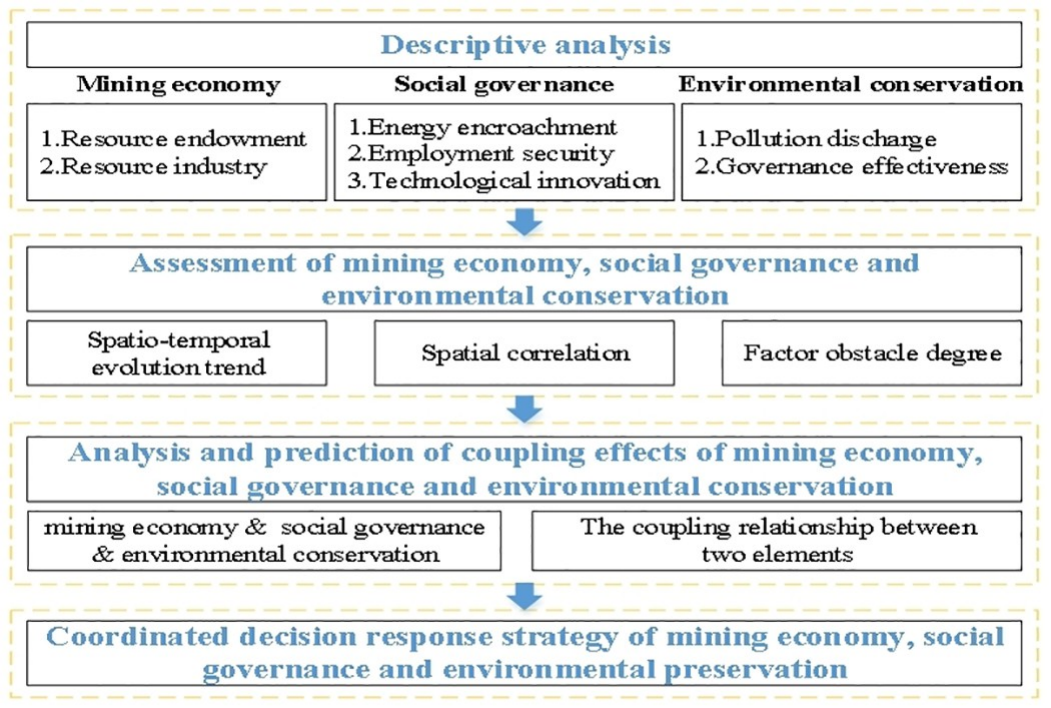
Frame diagram for the research.

To analyze the spatio-temporal heterogeneity and coupling effects of mining economy, social governance and environmental conservation, in this research, a *four-step method* is designed for the study, and Guangxi Zhuang Autonomous Region, a representative resource-rich region in China, is selected as an empirical analysis case. The specific process is as follows: firstly, descriptive analysis is used to sort out the status quo of mining economy, social governance and environmental conservation in the study area. Secondly, the related indicators of mining economy, society and environment are selected to construct the evaluation system and model, and the restrictive factors are identified by spatial analysis and barrier methods after the spatial-temporal dynamics quantitatively evaluated. Thirdly, based on the optimization coupling coordination effect analysis model, the coupling coordination state between mining economy, social governance and environmental conservation is measured. Finally, according to the above analysis conclusions, the idea of mining economy, society and environment collaborative governance is put forward (Fig 1).

### 2.2 Indicator system and model

#### 2.2.1 Indicator system

Influenced by research perspectives and data sources, objective indicators are often used to construct indicator system in previous studies on mining economy, but it is easy to lead to distortion of evaluation results. Therefore, this article follows the selection principle of the mining sector’s own indicators as the main, and the objective environmental constraint indicators as the auxiliary, and calculates some basic indicators to construct 23 index factors related to mining economy, social governance and environmental conservation [35, 36]. Detailed indicators, sources and attributes are shown in S1 Table, and *X*_1_, *X*_2_, ⋯ *X*_24_ indicates each indicator factor separately in the following sections.

#### 2.2.2 Evaluation of spatio-temporal heterogeneity

*(1) Evaluation model*. Mining economy, social governance and environmental conservation are the key factors that determine the sustainable development of resource-based cities, including resource endowment, industry, energy consumption, employment security, pollution emission and environmental governance. Based on the concept of comprehensive empowerment evaluation, we need to model it with subdivision indicators [15, 37]. According to the research idea, first of all, the development level of mining economy, social governance and environmental conservation needs to be comprehensively empowered and evaluated.


$$ME=\alpha_{1}X_{1}+\alpha_{2}X_{2}+\cdots +\alpha_{10}X_{11}$$ (1)

$$SG=\beta_{1}X_{12}+\beta_{2}X_{13}+\cdots +\beta_{7}X_{17}$$ (2)

$$EC=\gamma_{1}X_{18}+\gamma_{2}X_{19}+\cdots +\gamma_{7}X_{24}$$ (3)

In Eqs (1)–(3). *ME* refers to the development level of mining economy, *SG* refers to the development level of social governance, *EC* refers to the development level of environmental conservation. *α*,*β*,*γ* are the weights of corresponding indicator.

*(2) Entropy method*. It found that entropy method can avoid the influence of subjective factors, and it might use the information entropy relationship between data to determine the weight of indicators, so as to improve the accuracy of comprehensive evaluation results [38, 39].

First, positive or negative indicators are selected to standardize the data.

In Eq (4), positive indicators are as follows,

$$Y_{ij}=\frac{X_{ij}-Min(X_{j})}{Max(X_{j})-Min(X_{j})}(i=1,\ldots ,m;j=1,\ldots ,n)$$ (4)

In Eq (5), negative indicators are as follows,

$$Y_{ij}=\frac{Max(X_{j})-X_{ij}}{Max(X_{j})-Min(X_{j})}(i=1,\ldots ,m;j=1,\ldots ,n)$$ (5)

Suppose there are *m* cities, *n* indicators, and *X*_*ij*_ represents the original value of the indicator *j* for the city *i*.*Max(X*_*j*_*)* and *Min(X*_*j*_*)* respectively represent the maximum and minimum values of indicator j in all cities; *Y*_*ij*_ represents the value of index *j* of city *i* after dimensionless standardization, and the larger the *Y*_*ij*_, the greater the contribution to the target value (*Y*_*ij*_ ∈ [0,1]).

Then, the indicator weight should be determined. The proportion of the index value of object *i* under index *j* is shown in Eq (6).


$$p_{ij}=\frac{Y_{ij}}{\sum_{i=1}^{m}Y_{ij}}$$ (6)

The information entropy of index j is shown in Eq (7).


$$e_{j}=-\frac{1}{Inm}\sum_{i=1}^{m}p_{ij}Inp_{ij}$$ (7)

Finally, the weight of indicator *j* is determined as shown in Eq (8).


$$w_{j}=\frac{1-e_{j}}{\sum_{j=1}^{n}\left(1-e_{j}\right)}$$ (8)

In the above formulas, *i* = 1, …, *m*; *j* = 1, …, *n*.

#### 2.2.3 Coupling effect model

The coupling coordination degree is a measure of the interrelated effects between different systems [40]. Based on the evaluation results, the coupling effect model is used to identify the spatio-temporal changes of the interaction.


$$C={\left[\frac{\prod_{i=1}^{n}U_{i}}{\left(\frac{1}{n}\sum_{i=1}^{n}U_{i}\right)}\right]}^{\frac{1}{n}}(n=1,2,\ldots ,\infty )$$ (9)

$$T=\sum_{i=1}^{n}w_{i}U_{i}$$ (10)

$$D=\sqrt{C\times T}$$ (11)

In Eqs (1)–(3). *C* refers to coupling degree, *T* refers to comprehensive evaluation index, *D* refers to coupling coordination degree. *n* refers to number of indicators, *w*_*i*_ can be calculated using the entropy method, *U*_*i*_ refers to the value after evaluation. *C* ∈ [0,1], the larger the *C* value is, the higher the coupling degree will be. In addition, according to the actual research needs, this article divides the coupling effect into positive, general and negative states. The comparison of coupling coordination degree is shown in Table 1.

**Table 1:** Correspondence between coupling coordination degree and coupling state.

D value	Rank	Coupling degree	Coupling state
(0.0–0.1)	1	Extreme imbalance	Negative
[0.1–0.2)	2	Serious imbalance	
[0.2–0.3)	3	Moderate imbalance	
[0.3–0.4)	4	Mild imbalance	General
[0.4–0.5)	5	Borderline imbalance	
[0.5–0.6)	6	Forced coordination	
[0.6–0.7)	7	Primary coordination	
[0.7–0.8)	8	Intermediate coordination	Positive
[0.8–0.9)	9	Good coordination	
[0.9–1.0)	10	Quality coordination	

### 2.3 Measurement method

#### 2.3.1 Location quotient

Location quotient is a common spatial analysis method to judge the advantage of factors in resource production industries. By calculating the ratio between the share of a factor in the regional total and the share of the output value in the regional total output value, it can judge the superiority degree of the factor in the region [41]. This paper believes that the calculation of mineral resource reserves or resource industry advantages in a specific region is also applicable to location quotient.


$$RA_{j}=\frac{R_{j}/\sum_{i}R_{ij}}{\sum_{j}G_{j}/\sum_{i}\sum_{j}G_{ij}}-1$$ (12)

In Eq (12), *RA*_*j*_ refers to the preponderance of a certain resource or resource industry, *i* refers to Specific areas, *j* refers to a certain resource or resource industry, *R*_*j*_ refers to reserves or output value of the corresponding region, $\sum_{i}R_{ij}$ refers to the reserves or the output value of *j* resource or sector in the study area. $\sum_{j}G_{j}$ refers to GDP of the region, $\sum_{i}\sum_{j}G_{ij}$ refers to total GDP. The criteria for location advantage division are shown in Table 2.

**Table 2:** Regional advantage classification criteria.

Value	State
*RA*_*j*_ ≥ 1	Significant advantage
0.2 ≤ *RA*_*j*_ < 1	General advantage
0 ≤ *RA*_*j*_ < 0.2	Unstable advantage
-0.2 ≤ *RA*_*j*_ < 0	Unstable disadvantage
*RA*_*j*_ < -0.2	Disadvantage

#### 2.3.2 Input-output

Input-output is a commonly used industry correlation effect analysis method [42, 43]. In order to judge the correlation of resource industry sectors in the study area, reaction coefficient and influence coefficient are selected to reflect the interaction between sectors.

*(1) Input coefficient*.


$$a_{ij}=\frac{x_{ij}}{X_{j}}(i,j=1,2,\ldots ,n)$$ (13)

In Eq (13), *a*_*ij*_ refers to the amount of *i* sector directly consumed by the total output of *j* sector during production and operation, *x*_*ij*_ refers to the quantity of products or services directly consumed in the production and operation of *j* sector, *X*_*j*_ is the total input of *j* sector.

*(2) Leontief inverse matrix*.


$$B={\left(I-A\right)}^{-1}$$ (14)

$$A=\left[\begin{array}{cccc} a_{11} & a_{12} & \ldots & a_{1j}\\ a_{21} & a_{22} & \ldots & a_{2j}\\ \ldots & \ldots & \ldots & \ldots \\ a_{i1} & a_{i2} & \ldots & a_{ij} \end{array}\right]$$ (15)

In Eqs (14) and (15), *B* refers to Leontief inverse matrix, *I* refers to unit matrix, *A* refers to matrix of input coefficient.

(3) Reaction coefficient refers to the degree of demand induction received by a certain sector when each sector increases the final use of a unit, that is, the amount of output that the sector needs to provide for the production of other sectors. The larger the coefficient is, the stronger the role of the sector in promoting economic development.


$$E_{j}=\frac{\sum_{j=1}^{n}\bar{b_{ij}}}{\frac{1}{n}\sum_{i=q}^{n}\sum_{j=1}^{n}\bar{b_{ij}}}(i,j=1,2,\ldots ,n)$$ (16)

In Eq (16), *E*_*j*_ refers to reaction coefficient. $\sum_{j=1}^{n}\bar{b_{ij}}$ refers to the sum of rows *i* of Leontief’s inverse matrix, $\frac{1}{n}\sum_{i=q}^{n}\sum_{j=1}^{n}\bar{b_{ij}}$ is the average sum of the rows of the Leontief inverse matrix.

Influence coefficient refers to the degree to which the production demand of each sector is affected when one unit of final product is added to the sector. The larger the influence coefficient is, the stronger the pull effect of the sector on other sector is.


$$F_{j}=\frac{\sum_{i=1}^{n}\bar{b_{ij}}}{\frac{1}{n}\sum_{i=q}^{n}\sum_{j=1}^{n}\bar{b_{ij}}}(i,j=1,2,\ldots ,n)$$ (17)

In Eq (17), *F*_*j*_ refers to influence coefficient. $\sum_{i=1}^{n}\bar{b_{ij}}$ refers to the sum of columns *j* of Leontief’s inverse matrix, $\frac{1}{n}\sum_{i=q}^{n}\sum_{j=1}^{n}\bar{b_{ij}}$ is the average sum of the columns of the Leontief inverse matrix.

#### 2.3.3 Spatial autocorrelation

It found that spatial autocorrelation analysis can reflect the potential interdependence of observed variables in a region, which includes two measurement methods: global spatial autocorrelation and local spatial autocorrelation [44, 45]. In this paper, *Moran’s I* and *Lisa* are used to analyze the spatial correlation characteristics of mining economy, social governance and environmental conservation.

*(1) Global spatial autocorrelation*.


$$MoranI=\frac{m}{\sum_{i=1}^{m}\sum_{j=1}^{m}w_{ij}}\times \frac{\sum_{i=1}^{m}\sum_{j=1}^{m}w_{ij}(S_{i}-\bar{S})(S_{j}-\bar{S})}{\sum_{i=1}^{m}{\left(S_{j}-\bar{S}\right)}^{2}}$$ (18)

In Eq (18), *Moran’sI* refers to the value of global spatial autocorrelation, and its exponent ranges from [−1,1]. *w*_*ij*_ refers to the spatial weight matrix in the region, that is, take 1 when *i* is adjacent to *j*, and 0 otherwise. $\bar{S}$ is the average of *S*_*i*_. In addition, if the value is greater than 0, the correlation is positive, and the larger the value is, the stronger the spatial aggregation is; If the value is less than 0, the correlation is positive is negative, and the smaller the value is, the stronger the spatial difference is; The value equal to 0 indicates that the space is uncorrelated and the spatial units are randomly distributed.

*(2) Local spatial autocorrelation*.


$$I_{i}=\frac{m(S_{i}-\bar{S})\sum_{j=1}^{m}w_{ij}(S_{j}-\bar{S})}{\sum_{i=1}^{m}{\left(S_{i}-\bar{S}\right)}^{2}}$$ (19)

In Eq (19), *I*_*i*_ refers to the value of local spatial autocorrelation, and its exponent ranges from [−1,1]. Positive value indicates that the attribute values of spatial unit *i* are similar to those of neighboring regional units, and negative value indicates spatial differentiation.

#### 2.3.4 Factor barrier degree

The factor barrier degree model is mainly to investigate the influence of each indicator of the factor layer on the target, so as to identify the constraints affecting the mining economy, social governance and environmental conservation [46].


$$O_{ij}=1-Y_{ij}$$ (20)

$$z_{ij}=\frac{w_{j}\times O_{ij}}{\sum_{j=1}^{n}w_{j}\times O_{ij}}\times 100\%$$ (21)

In Eqs (20) and (21), *O*_*ij*_ refers to the deviation degree of the indicator, *Z*_*ij*_ refers to the obstacle degree of *j* indicator of city *i*. In addition, based on the barrier degree score of each indicator, the barrier degree score of each factor in the factor layer can be obtained by adding up the barrier degree score according to the belonging relationship.

### 2.4 Data sources

The data used in this study are composed of panel data related to mining economy, social governance and environmental conservation in Guangxi Zhuang Autonomous Region of China from 2010 to 2021. These data are mainly gathered from the World Bank, China City Statistical Yearbook, Guangxi Statistical Yearbook, Guangxi Mineral Resources Statistical Bulletin, Guangxi Ecological and Environmental Protection Bulletin, China Emission Accounts and Datasets, Guangxi 42 departments input-output tables. In addition, the relevant data of mineral origin, resource distribution and reserves in Guangxi are derived from Chinese Geology and Mineral Resources in Guangxi Volume. The partly calculated results can be found in S2 and S3 Tables, and some missing data is supplemented by data fitting method.

## 3. Empirical analysis

### 3.1 Overview of mining economy, social governance and enviro-nmental conservation in Guangxi

#### 3.1.1 Mining economy

**Fig 2:**
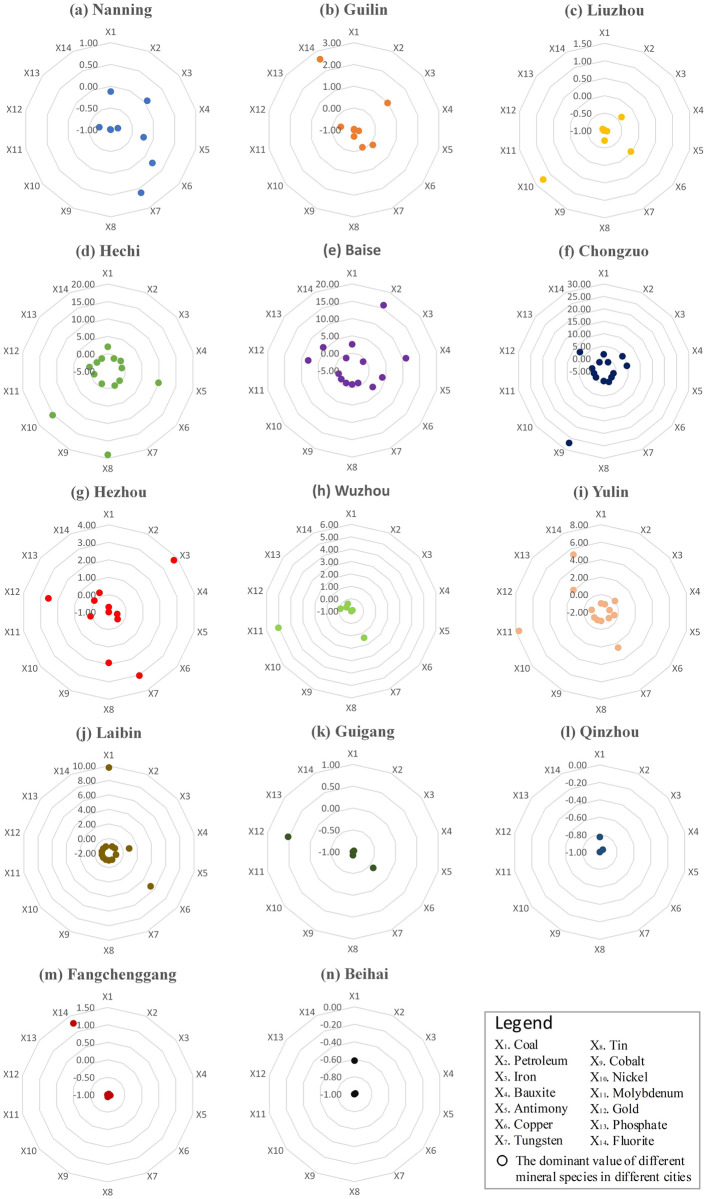
Spatial difference of location advantage of main minerals in Guangxi.

As for the mineral endowments. There are 13 kinds of mineral resources with significant advantages in Guangxi, among which the metallic ores are tin, manganese, bauxite, antimony, zinc, tungsten and pyrite, the non-metallic ores are kaolin, bentonite, barite, marble, talc and limestone, and the minerals with general advantages are gold, vanadium and granite. Nickel ore presents an unstable advantage, and other minerals such as petroleum, coal and iron have significant disadvantages (Table 3). The regional distribution characteristics of the 14 strategic critical minerals that meet the recoverable criteria are shown in Fig 2. Among the energy resources, only the coal resources of Hechi, Baise and Laibin have comparative advantages, a small amount of oil can be seen in Baise city, and there is no recoverable storage of natural gas and shale gas resources. Among the non-energy resources, Guilin only have a comparative advantage in fluorite resources; Liuzhou only have a comparative advantage in nickel resources; Laibin only have comparative advantage in copper resources; Hechi has comparative advantages in antimony, tin and nickel resources; Baise has comparative advantages in bauxite, antimony, copper, gold and phosphate resources; Chongzuo has comparative advantages in iron, bauxite, cobalt and phosphate resources; Hezhou has comparative advantages in iron, tungsten, tin and gold resources; Wuzhou has comparative advantages in tungsten and molybdenum resources; Yulin has comparative advantages in tungsten, molybdenum, phosphate and fluorite resources; Fangchenggang only have a comparative advantage in fluorite resources; Nanning, Guigang, Qinzhou and Beihai have no significant advantages in the endowment of key mineral resources. In general, the overall mineral resource endowment advantages of Hechi, Baise, Chongzuo, Hezhou, Wuzhou and Yilin are outstanding, and their key mineral reserves have comparative advantages in Guangxi. Moreover, the calculation results of the superiority degree of resource endowments are shown in S2 Table.

**Table 3:** Overall situation of mineral resource reserve advantage in Guangxi.

Mineral	Value	Status	Mineral	Value	Status
tin	15.9	Significant advantage	nickel	0.02	Unstable advantage
kaolin	14.19	Significant advantage	titanium	-0.21	Disadvantage
manganese	10.46	Significant advantage	gesso	-0.29	Disadvantage
bentonite	10	Significant advantage	fireclay	-0.32	Disadvantage
bauxite	8.47	Significant advantage	fluorite	-0.34	Disadvantage
barite	7.97	Significant advantage	silver	-0.37	Disadvantage
antimony	6.04	Significant advantage	lead	-0.44	Disadvantage
marble	4.13	Significant advantage	wollastonite	-0.49	Disadvantage
talc	3.78	Significant advantage	copper	-0.68	Disadvantage
zinc	2.37	Significant advantage	iron	-0.71	Disadvantage
tungsten	1.63	Significant advantage	petroleum	-0.77	Disadvantage
limestone	1.55	Significant advantage	coal	-0.92	Disadvantage
pyrite	1.45	Significant advantage	Phosphate	-0.93	Disadvantage
gold	0.83	General advantage	mirabilite	-0.95	Disadvantage
granite	0.81	General advantage	cobalt	-0.96	Disadvantage
vanadium	0.57	General advantage	molybdenum	-0.96	Disadvantage

**Fig 3:**
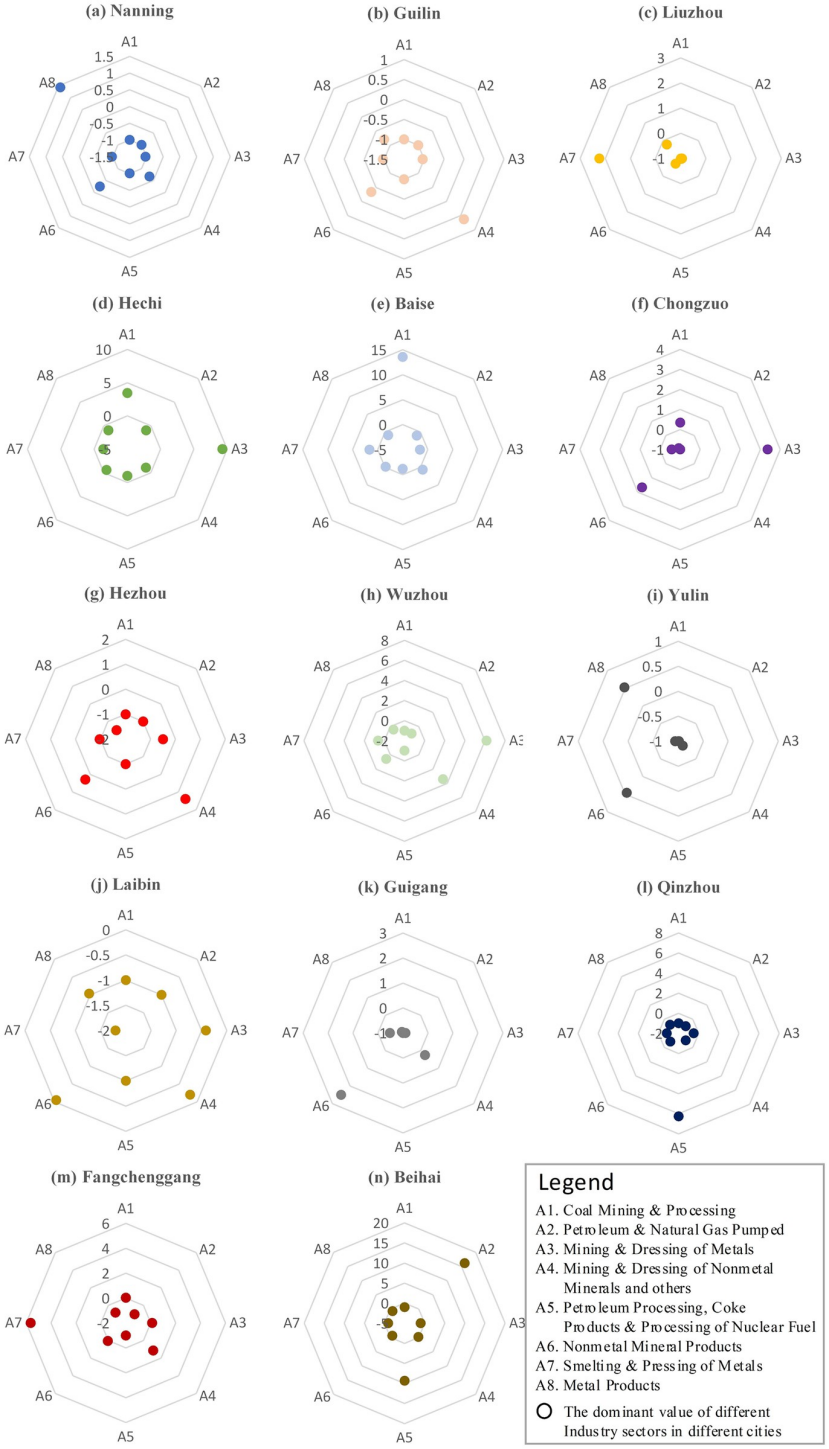
Spatial difference of location advantage of industry sectors in Guangxi.

As for the comparative advantage of resource industry. On the whole, the sector of *Nonmetal Mineral Products* has significant comparative advantages, the sectors of *Mining & Dressing of Nonmetal Minerals and others*, *Petroleum Processing*, *Coke Products & Processing of Nuclear Fuel* and *Smelting & Pressing of Metals* have general advantages, the sector of *Mining & Dressing of Metals* has unstable comparative advantages, and the disadvantage position is reflected in *Coal Mining & Processing*, *Petroleum & Natural Gas Pumped* and *Metal Products* sectors (Table 4). In addition, the location advantage of resource industry is closely related to the conditions of resource endowment. and spatial difference of location advantage of Guangxi’s industrial sectors are shown in Fig 3. Hechi and Baise have comparative advantages in the sectors of *Coal Mining & Processing* and *Mining & Dressing of Metals*; Nanning, Liuzhou and other regions with good economic foundation mainly undertake the deep processing of resource products, and their sectors of *Smelting & Pressing of Metals* and *Metal Products* have significant comparative advantages; Qinzhou and Beihai have significant comparative advantages in the sectors of *Petroleum & Natural Gas Pumped* and *Petroleum Processing*, *Coke Products & Processing of Nuclear Fuel* due to their good petrochemical industry foundation; The distribution of mineral resources in Guilin, Wuzhou, Fangchenggang and other regions is disorderly, which leads to the relatively random comparative advantages of their resource industry sectors. Moreover, the calculation results of the superiority degree of resource industrial sectors are shown in S3 Table.

**Table 4:** Comparative advantage of resources industry sectors in Guangxi.

Sectors	Value	Status
A6	1.8	Significant advantage
A7	0.45	General advantage
A4	0.4	General advantage
A5	0.26	General advantage
A3	0.04	Unstable advantage
A2	-0.65	Disadvantage
A8	-0.69	Disadvantage
A1	-1	Disadvantage

In Table 4, A_1_ is Coal Mining & Processing, A_2_ is Petroleum & Natural Gas Pumped, A_3_ is Mining & Dressing of Metals, A_4_ is Mining & Dressing of Nonmetal Minerals and others, A_5_ is Petroleum Processing, Coke Products & Processing of Nuclear Fuel, A_6_ is Nonmetal Mineral Products, A_7_ is Smelting & Pressing of Metals, A_8_ is Metal Products.

As for the correlation effect of industry. The changes of reaction and influence coefficient among resource industry sectors from 2007 to 2017 are shown in Table 5, and some conclusions can be drawn after input-output analysis. (1) Coal is not the dominant mineral in Guangxi, and only a few of resources exist in Hechi and Baise, which led to a reduction in the supply and demand intensity of *Coal Mining & Processing* to other industrial sectors; (2) Although the foundation of *Petroleum & Natural Gas Pumped* in Guangxi is weak, as the key energy for national economic security, it is closely related to other product sectors; (3) The adjustment of the mining policy led to the gradual contraction of *Mining & Dressing of Metals* after 2012, and its material demand intensity for other sectors weakened, but the material demand intensity of the downstream industry for this sector was relatively balanced; (4) The trend of *Mining & Dressing of Nonmetal Minerals and others* is basically the same as that of *Mining & Dressing of Metals*, but the development of surrounding industries such as calcium carbonate and decorative materials has promoted the consumption of non-metallic ore resources in Guangxi; (5) Guangxi has a high degree of external dependence on oil and natural gas, and the limited scale of *Petroleum Processing*, *Coke Products & Processing of Nuclear Fuel* led to a decrease in the intensity of material supply to other industrial sectors. However, the industrial expansion in Qinzhou and Beihai requires a large number of production materials supplied by other industrial sectors; (6) *Nonmetal Mineral Products* in Guangxi has a weak ability to promote other industrial sectors, but the production activities of this sector have a high material demand intensity for other sectors. (7) As a traditional dominant industry in Guangxi, *Smelting & Pressing of Metals* has a high degree of reaction coefficient and influence coefficient. However, with the change of mining policy, the material demand intensity of this sector to other industrial sectors has weakened. (8) The reaction and influence coefficient of *Metal Products* also have reduced due to the downstream industry of *Smelting & Pressing of Metals*. On the whole, the correlation effect between the mining and processing industry of mineral resources and other industrial sectors continues to weaken, but it is still in a dominant position. The correlation effect between the relevant departments of superior minerals and other industrial departments is still at a high level, but with the change of the resource industry structure, the advantages of the surrounding industries of non-ferrous metals are gradually transferred to the non-metallic industries, and oil and gas related industries are in a state of scale expansion.

**Table 5:** Reaction coefficient and influence coefficient of resource industry sectors.

Sectors	2007		2012		2017	
	Reaction	Influence	Reaction	Influence	Reaction	Influence
A_1_	1.14	0.92	1.07	0.90	0.91	0.81
A_2_	0.91	0.47	1.19	0.44	1.1	0.75
A_3_	1.05	0.91	1.17	0.91	0.89	0.95
A_4_	0.61	0.87	0.66	0.89	0.65	1.01
A_5_	1.46	0.78	1.39	0.90	1.29	1.00
A_6_	0.78	1.22	0.81	1.12	0.76	1.12
A_7_	2.48	1.26	2.63	1.21	0.74	1.19
A_8_	0.92	1.24	0.76	1.22	0.74	1.19

#### 3.1.2 Social governance

**Fig 4:**
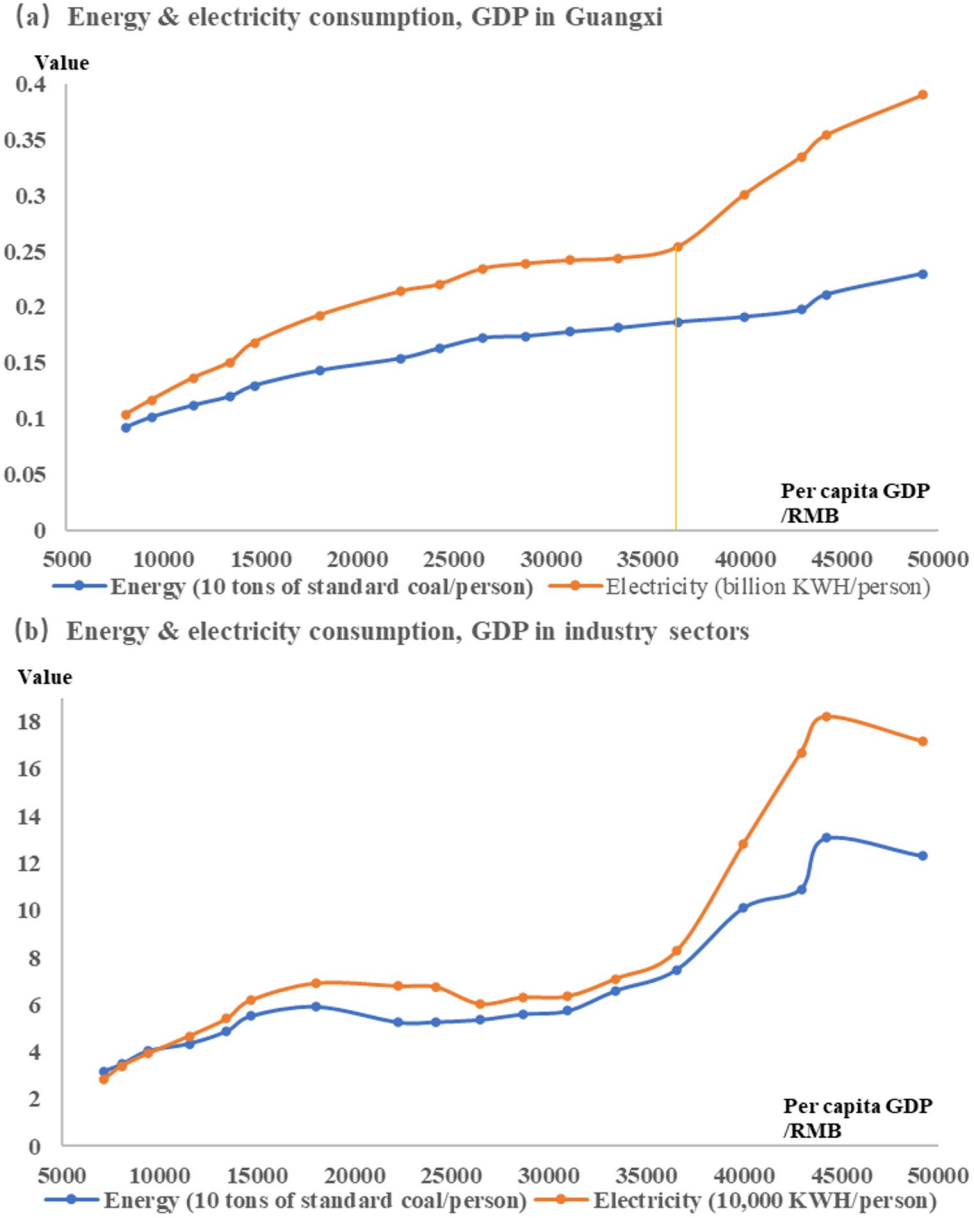
The relationship between energy & electricity consumption, GDP.

As for energy resource encroachment. Due to the limitation of resource endowment structure, the external dependence of energy and electricity consumption in Guangxi is high. The per capita consumption of energy and electricity and per capita GDP in Guangxi have a significant positive promoting relationship, that is, increasing energy and electricity consumption can effectively increase per capita GDP. The trajectory of energy, electricity consumption and per capita GDP in the resource industry sector is the same as that of the whole region, but the growth rate is different (Fig 4). According to the limit theory of the consumption growth of bulk energy and mineral resources [47, 48], this article believes that the consumption of energy and electricity also conforms to the "S-shaped" curve trajectory. With per capita GDP of 10,000 yuan as the starting point of energy and power consumption input, per capita energy and power consumption is at the take-off point when it is 30,000 to 40,000, that is, with the further investment in energy and power consumption, per capita GDP will grow slowly and eventually reach the limit of energy and power consumption. It shows that the current energy and electricity consumption of the resource industry sector has not reached the peak, and the effect of social resource appropriation will be further enhanced, but the per capita GDP growth rate shows a downward trend.

**Fig 5:**
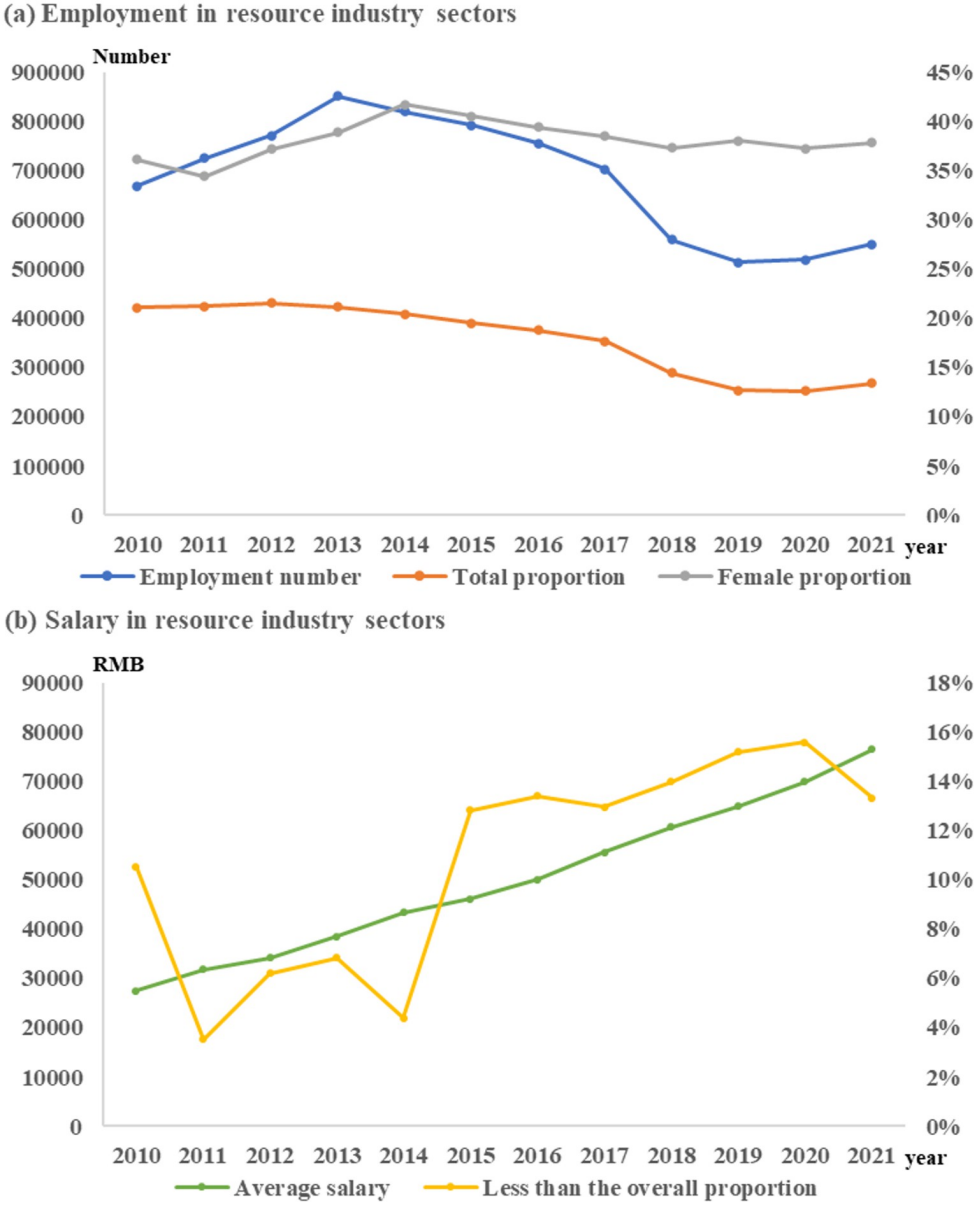
Employment guarantee in resources industry sectors of Guangxi from 2010 to 2021.

As for the Employment security dilemma. Before 2013, the number of employments absorbed by the resource industry in Guangxi continued to rise, which was the highest in the "12th Five-Year Plan" and "13th Five-Year Plan" period. From 2013 to 2021, the employment absorption ability of the resource industry sector continued to decrease, falling by 32.8% compared with 2010. The employment capacity of urban units continues to rise, but the proportion of resources industry in total employment is declining. Among them, the number of women engaged in resource production activities was basically the same as that at the beginning of the "Twelfth Five-Year Plan", with an increase of only 4.8%. The average wage of the resource industry sector increased by 178.5% compared with the early period of the "Twelfth Five-Year Plan", the growth rate is lower than the average wage increase. The average wage level of resource industry sector is lower than the regional average wage, the overall gap is more than 10%, and only in some years, the wage gap is less than 10% (Fig 5). It shows that the employment security capacity of the resource industry sector in Guangxi is an important part of the employment security system, but the employment absorption capacity is in the process of weakening. The wage level of the resource industry sector increases with the regional economic growth, but it is far lower than that of other industrial sectors in the same period.

As for the intensity of technological innovation. From 2010 to 2021, the number of R&D full-time employees in Guangxi’s resources industry continued to rise, which was 839.6% higher than the initial period of the "12th Five-Year Plan", and accounted for 27.2% of the total number of regional E&D full-time employees. The R&D expenditure of the resource industry is ten times that of the beginning of the 12th Five-Year Plan, and its proportion in the total investment has increased by more than 20%. Among them, the research expenditure has slightly increased, and the proportion of experimental development expenditure has significantly increased (Table 6). It shows that with the transformation of economic structure, the progress of transformation and development of the resource industry has been accelerated, the relevant technology improvement work for the resource industry has been continuously promoted, the research and practice process in the field of resource industry has been deepened, and the external environmental factors affecting the quality of industrial development have improved, but they are still at a low level compared with other provinces in China.

**Table 6:** Investment intensity of R&D personnel and funds in resource industry sectors of Guangxi from 2010 to 2021 (Ten thousand yuan).

Year	Personnel	Proportion	Funds	Proportion	Research	Development
2010	824	5.5%	30762.3	6.5%	0.2%	6.4%
2011	1793	10.6%	74630	15.0%	1.0%	14.0%
2012	2369	12.8%	98196.6	16.1%	0.1%	16.0%
2013	2287	12.9%	159347.3	22.2%	0.1%	22.2%
2014	1912	9.5%	181214.7	24.3%	0.1%	24.2%
2015	1573	9.7%	99995.3	15.0%	0.1%	15.0%
2016	1401	8.8%	121818	17.2%	0.4%	16.9%
2017	1303	9.8%	156007.9	19.2%	0.1%	19.1%
2018	2166	12.6%	238236.9	26.7%	0.3%	34.4%
2019	3621	16.4%	312961.4	30.0%	0.3%	29.7%
2020	3703	18.2%	273326.6	24.1%	0.2%	25.9%
2021	7742	27.2%	296237.5	21.6%	0.2%	23.2%

#### 3.1.3 Environmental conservation

In general, China aims to achieve "carbon peak" and "carbon neutral" goals by 2030 and 2060. Resource industry is a highly polluting industry, and its pollution control effect determines the level of environmental conservation.

**Fig 6:**
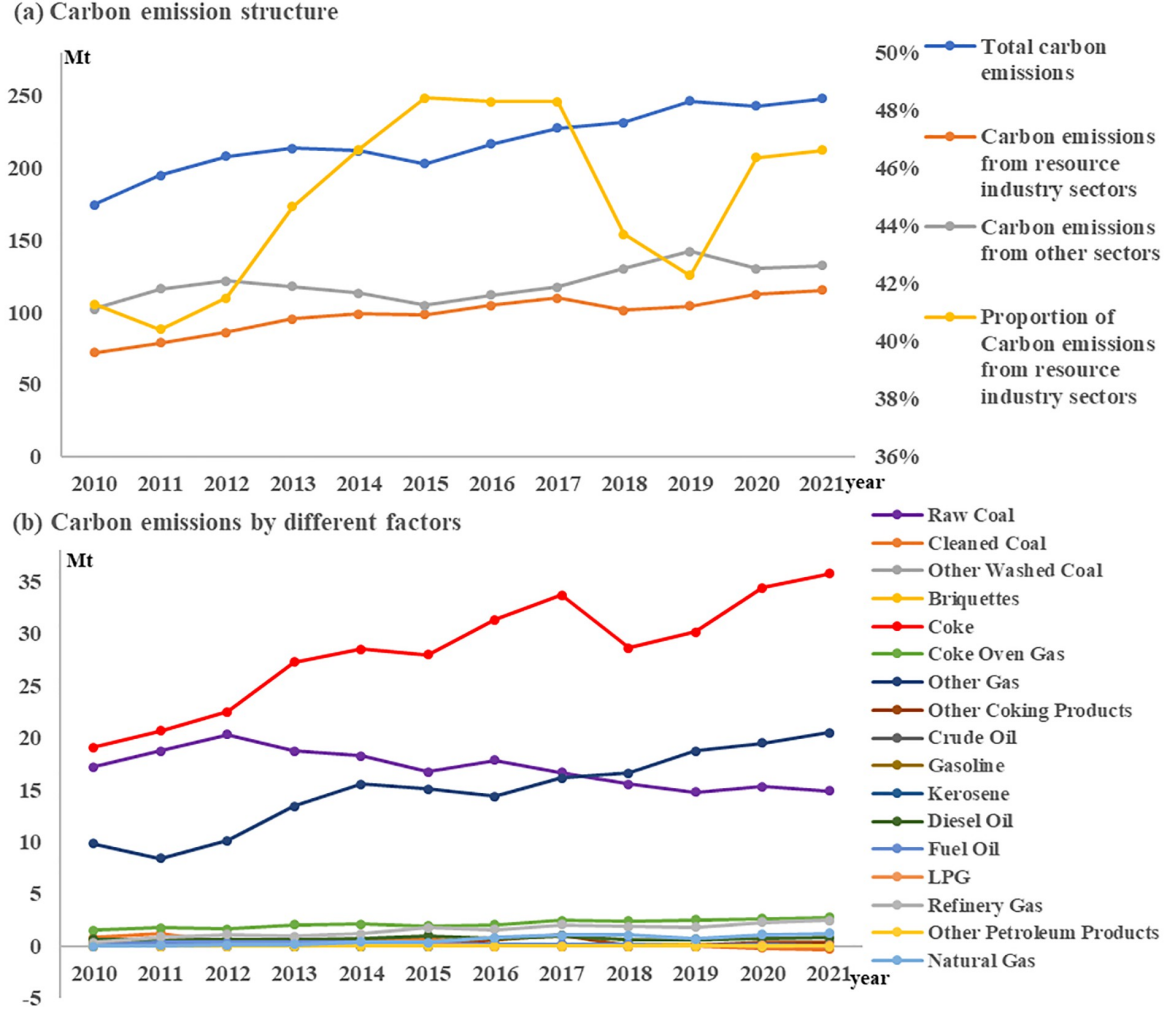
Carbon emission structure and emission factors.

As for pollution emissions. Influenced by the favorable market, the carbon emissions of the resource industry rose weakly in the short term from 2010 to 2012; The continuous tightening of environmental policy from 2012 to 2015 led to the reduction of carbon emissions; From 2015 to 2021, the transformation of the resource industry achieved results, and the carbon emissions showed an obvious upward trend. However, due to the influence of the overall requirements of social and environmental governance, the carbon emission intensity gradually weakened after 2017, and the carbon emission structure changed significantly. This shows that the resource industry sectors are important sources of carbon emission pollution in Guangxi, and the environmental pollution caused by the production and operation activities of the resource industry sectors are still serious. Coke, raw coal, and other gases were the key factors leading to carbon emission pollution in the resource industry sectors. Specifically, influenced by the changes in the energy consumption structure of the resource industry, the carbon emissions caused by coke and other gases continued to rise, while the carbon emissions caused by raw coal showed a downward trend (Fig 6). It shows that the key to curb the carbon emission pollution of the resource industry in Guangxi is to further promote the transformation of energy consumption structure and gradually reduce the consumption of coke, raw coal and other gas energy resources.

As for environmental investment. The investment in industrial pollution control in Guangxi was significantly different, and the mining market and policy changes had a great impact on the investment in pollution control, with the proportion of investment in pollution control exceeding 10% in 2011, 2015 and 2020 (Table 7). Combined with the analysis of regional carbon emission data, the investment tendency of Guangxi’s regional environmental governance was in other fields except the production activities of the resource industry, and the pressure of environmental pollution caused by the production activities of the resource industry was still large. In terms of the governance effect, except for 2015 and 2017, the proportion of air quality above the excellent level in other years was more than 90%, indicating that high investment can play a positive role in promoting environmental conservation.

**Table 7:** Pollution control investment and effectiveness.

Year	Pollution control		Effectiveness
	Investment in industrial	Proportion	Days of good air
	(Ten thousand yuan)		
2010	92845	4.5%	92.9%
2011	472799	21.3%	98.8%
2012	127329	6.4%	98.8%
2013	183218	8.4%	95.8%
2014	178909	8.7%	95.6%
2015	247151	10.6%	88.5%
2016	130433	6.8%	93.5%
2017	75847	3.8%	88.5%
2018	58273	4.8%	91.6%
2019	48620	4.1%	91.7%
2020	35535	10.2%	97.7%
2021	119600	5.8%	95.8%

#### 3.1.4 Basic situation judgment

First of all, from the perspective of mining economy, some mineral resource endowments in Guangxi still have significant comparative advantages in the whole country, and the spatial distribution of typical mineral resources in the region has changed, but it is in a relatively stable situation. Due to the particularity of the advantages of the resource industry, the competitive advantages of some traditional industrial sectors have weakened, and the balanced development of the resource industry has gradually formed.

Then, from the perspective of social governance, the intensity of energy consumption of the resource industry sector is still rising, and as a high-energy industry sector, it has brought great adjustment to social governance. Although the wage level of the resource industry sector has increased, its driving ability for social employment has been weakening. In addition, due to the influence of industrial transformation policies, the scientific and technological innovation intensity of the resource industry sector is relatively high.

Finally, from the perspective of environmental conservation, the main carbon emission factors of the resource industry are basically stable. Although the investment in environmental conservation is constantly increasing, the total emissions are still rising at a low speed. However, by comparing with the policies of different time periods, we found that the industrial, environmental and capital policies had a great impact on the environmental pollution of the resource industry.

It can be seen that the restrictive relationship among mining economy, social governance and environmental conservation is in a relatively balanced state. Although Guangxi is China’s non-ferrous metal resources and industrial advantages, it is facing great economic, social and environmental pressure at the present stage, and it needs to systematically promote the coupling effect of the three.

### 3.2 Spatio-temporal heterogeneity of mining economy, social gov-ernance and environmental conservation

#### 3.2.1 Assessment of development level

This article collected the panel data related to mining economy, social governance and environmental conservation in Guangxi from 2011 to 2021, and used the weighted comprehensive evaluation method to measure the spatio-temporal differences in the development level of the whole region and 14 cities. Here, the natural break point (Jenks) was used to investigate the spatial heterogeneity of each city in 2021, and the levels were divided into high, medium and low.

**Fig 7:**
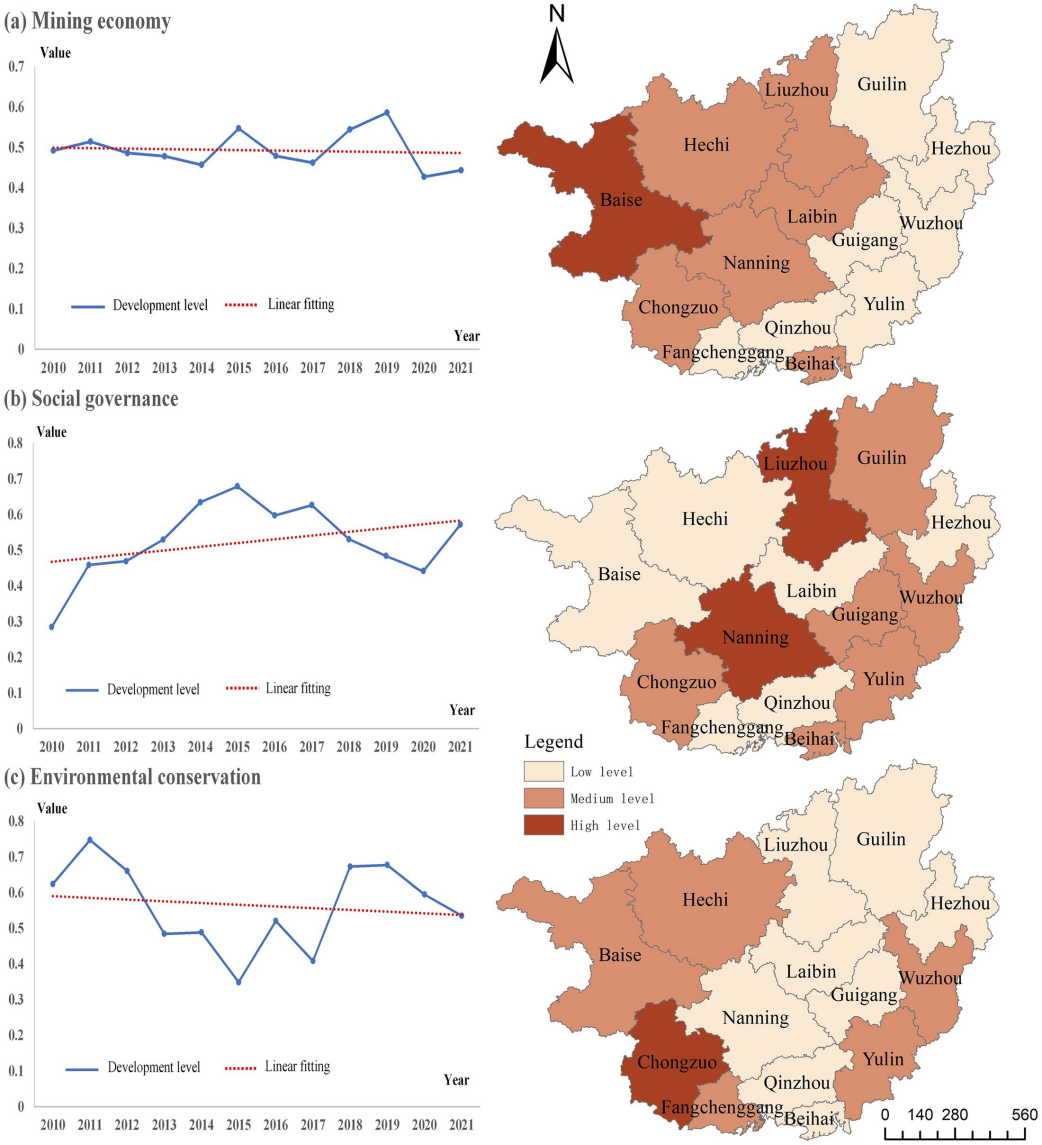
Assessment of mining economy, social governance and environmental conservation.

From the analysis of Fig 7, it can be found that the levels of mining economy, social governance and environmental conservation in Guangxi from 2010 to 2021 have obvious spatial and temporal heterogeneity. From the perspective of mining economy, the supporting effect of resource industry on regional economy was basically stable, and there were weak dynamic fluctuations only in some years. From the perspective of social governance level, although it has declined after 2015, the social security capacity of the resource industry has shown an overall upward trend. From the perspective of environmental conservation level, before 2017, the resource industry had a greater role in damaging the regional environment, and after that, although the environmental preservation level has improved, it has generally shown a downward trend. It shows that the key position of Guangxi’s resource industry economy still exists, and its driving effect on social governance is obvious. Although the tightening of resource and environmental policy links the environmental damage of resource industry, its inhibitory effect is limited.

The spatial distribution of mining economy, social governance and environmental conservation is relatively random, but there is a certain internal logical correlation (Fig 7). (1) The advantages of mining economy in the west of Guangxi were greater than those in the east of Guangxi, which indicated that the development level of mining economy was closely related to resource endowment and industrial base. The mining economy level was the highest in areas with superior resource endowment, such as Baise, Hechi, Chongzuo and Laibin. Nanning, Liuzhou and other central cities had obvious economic advantages, good deep processing capacity of resource products, and their mining economic level was relatively high. In other regions, the resource and industrial base were relatively weak, and the economic advantages of mining were not obvious. (2) Due to the differences in the impact of resource industry chains on social governance, the spatial distribution of social governance was relatively random. The resource siphon effect of central cities led to their strong social governance capacity. The resource exploration and development industries were located in areas with advantages in resource endowment, and the energy encroachment effect in such areas was strong, but the driving effect on employment was limited. However, the smelting, processing and circulation links of resource products belong to labor-intensive industries, and the social security ability of such areas was relatively stronger. (3) Affected by resource reserves and environmental regulation policies, traditional industries in regions with superior resource endowments lost their first-step advantages. Environmental governance was an important issue faced by such regions, and greater human and material resources needed to be invested to solve the environmental damage caused by the development of resource industries. At the same time, other cities were less constrained by the policy, and their pollution emission intensity and environmental governance investment decreased accordingly.

#### 3.2.2 Analysis of influencing factors

The assessment results of development level only show that Guangxi mining economy, social governance and environmental conservation have significant spatial and temporal differences. In order to reveal the spatial correlation effect of each factor, Moran I and Lisa index are calculated by spatial autocorrelation analysis. In addition, the factor barrier degree is used to identify the restrictive indicators.

**Fig 8:**
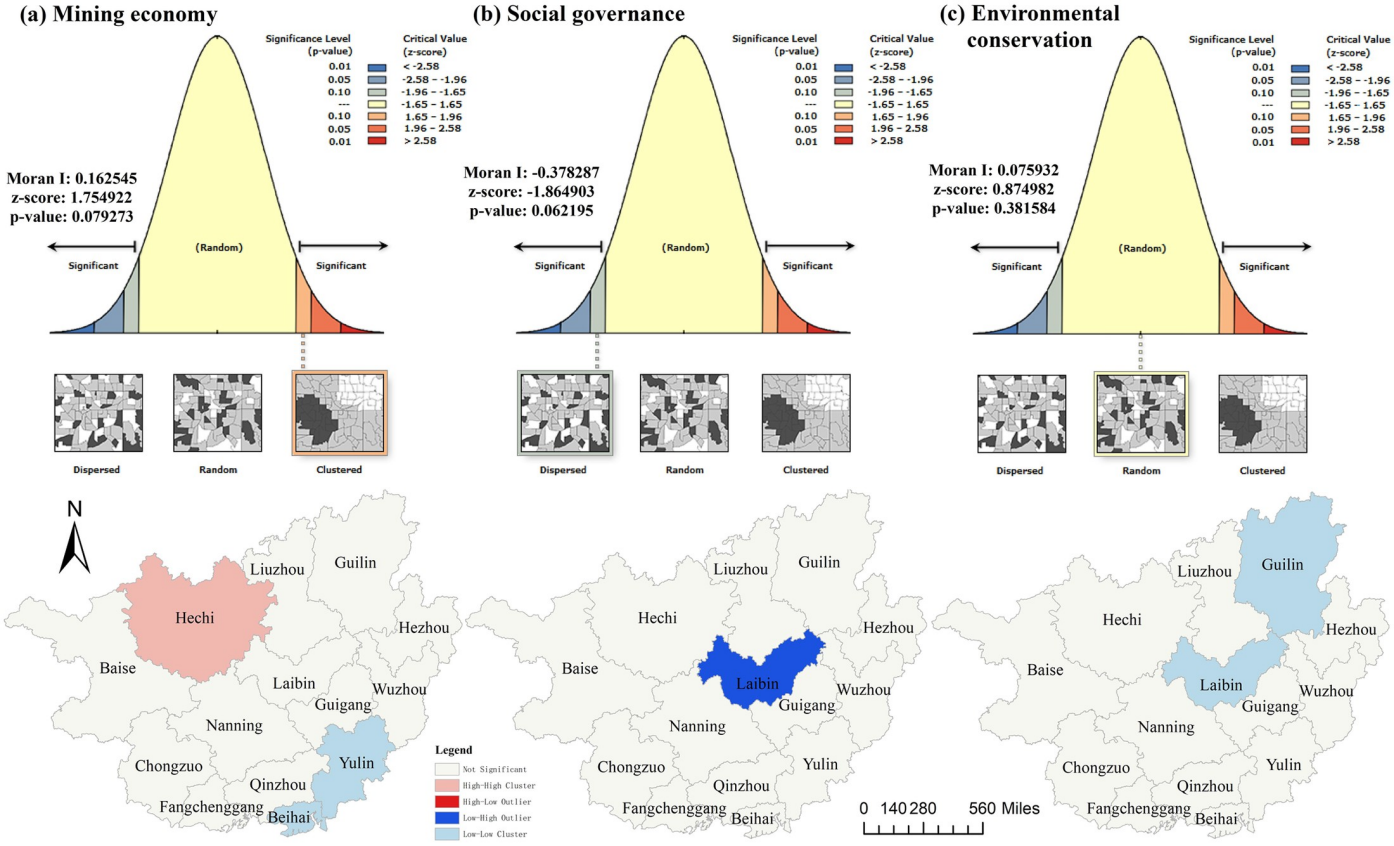
Spatial distribution characteristics of mining economy, social governance and environmental conservation.

*(1) Spatial autocorrelation*. It shows the global autocorrelation report and Lisa diagram drawn by spatial autocorrelation simulation using ARCGIS software (Fig 8). From the perspective of global autocorrelation, the Moran I index of mining economy is greater than 0, and the simulation results show that it is in the cluster distribution interval, which indicates that the mining economy in Guangxi presents spatial aggregation effect, that is, the mining economic index is positively correlated with regional aggregation degree. The Moran I index of social governance is less than 0, and the simulation results show that it is in the discrete distribution interval, which indicates that the social governance in Guangxi presents spatial discrete effect, that is, the social governance index is negatively correlated with the regional aggregation degree. In addition, since the Moran I index of environmental conservation is close to zero and fails the p-value test, it indicates that the distribution of environmental conservation is relatively random. The local autocorrelation outlier test method is further used to analyze the specific situation of spatial clustering in each dimension. In the mining economic system, Beihai and Yulin show low-low aggregation; The local autocorrelation effect is weak in the social governance system; In the environment conservation system, Guilin and guests present low—low aggregation. This shows that the spatial correlation effect of mining economy, social governance and environmental conservation in Guangxi is weak, and the spatial distribution of each factor governance is relatively random.

**Fig 9:**
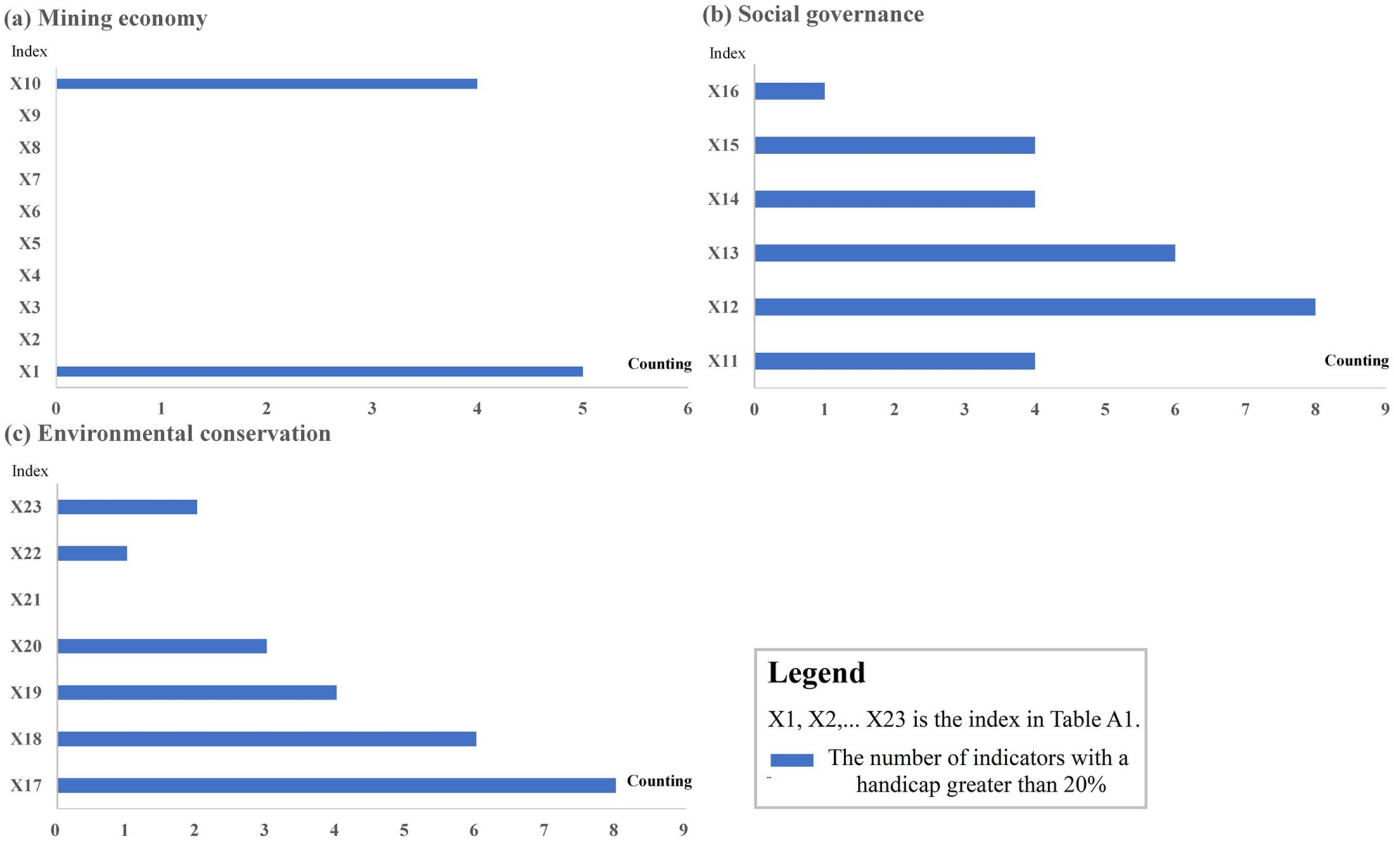
Obstacle frequency distribution of the indicators.

*(2) Factor barrier degree*. Based on the standardized data of Guangxi Mining economy, social governance and environmental conservation from 2010 to 2021, this article uses the obstacle degree analysis method to calculate. Combined with the research needs and the actual calculation results, the obstacle degree greater than 20% is defined as the limiting factor, and the number of factors that meet the standards is counted (Fig 9). It is found that in the mining economic system, the proportion of medium-sized or above mineral producing areas and the proportion of secondary and tertiary industry structure have the highest obstacle degree of more than 20%, which indicates that the resource guarantee ability and industrial structure have a high impact on the mining economy. In the social governance system, the number of employees and average wages in the resource industry are the indicators with the largest number of obstacles exceeding 20%, indicating that the social security role of the resource industry is mainly reflected in employment. Finally, in the environmental conservation system, the largest number of indicators with barrier degree exceeding 20% are carbon emission intensity and wastewater discharge from resource industry sectors, indicating that CO2 and industrial wastewater are key factors affecting environmental conservation.

### 3.3 Coupling coordination effect among mining economy, social governance and environmental conservation

**Fig 10:**
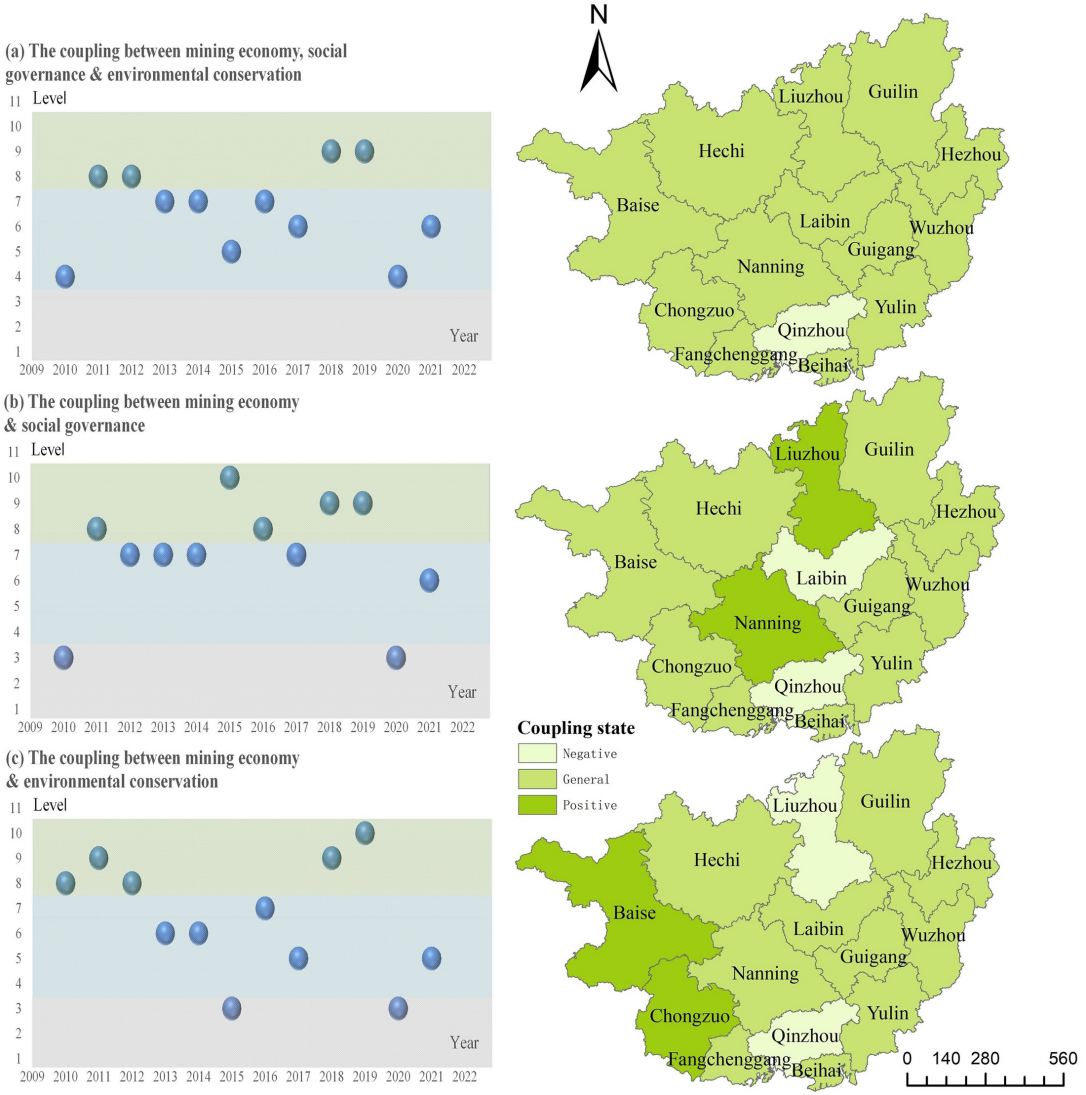
Spatio-temporal heterogeneity of the coupling effect among mining economy, social governance and environmental conservation.

The above analysis is a preliminary study on the spatio-temporal heterogeneity and constraints of mining economy, social governance and environmental conservation in Guangxi. It should be noted that coupling coordination is an important goal for the sustainable development of resource-based cities. Therefore, this article uses the coupling effect model to measure the coupling relationship between elements. The calculation results of coupling coordination degree are shown in Fig 10.

The study finds that the coupling effect between mining economy, social governance and environmental conservation from 2010 to 2021 showed great uncertainty, but the overall coupling state was above general. From the perspective of the coupling effect among mining economy, social governance and environmental conservation, except for 2010, 2017 and 2020, the other years were all at the level of barely coordination, among which 2011, 2012, 2018 and 2019 were at the level of intermediate coordination. The overall level of coupling effect between mining economy and social governance was high, and except for 2010, 2020 and 2021, other years were above the intermediate coordination level. The coupling effect between mining economy and environmental maintenance was relatively poor, and it was below the level of near imbalance in some years. It showed that mining economy could promote social governance, but affected by environmental policies, mining economy and environmental conservation were negatively correlated, and the coupling effect between mining economy, social governance and environmental conservation needed to be further improved.

In addition, this article selected the cross-section data of mining economy, social governance and environmental conservation index of 14 cities in 2021 to investigate the spatial distribution of coupling effect among elements. Except Qinzhou, the coupling effect of mining economy, social governance and environmental conservation in the other 13 cities was in the general state. The coupling effect of mining economy and social governance in central cities such as Nanning and Liuzhou were in the negative state, Qinzhou and Laibin were in the positive state, and the other cities were in the general state. The coupling effect between mining economy and environment conservation was strong in Baise, Chongzuo, Fangchenggang and other resource-endowed regions. Qinzhou and Liuzhou were in a positive state, while other cities are in a general state. It shows that the regional differences of mining economy, social governance and environmental maintenance lead to the random distribution of their coupling effect, and the coordinated governance situation of regional elements has not yet formed. The coupling and coordination of mining economy and social governance needs to rely on a good economic and industrial foundation. Constrained by resource and environmental policies, regions with superior resource endowments need to promote resource industry transformation and environmental governance simultaneously, resulting in higher coupling and coordination between mining economy and environment conservation.

## 4. Conclusions and discussion

### 4.1 Conclusions

Through in-depth analysis of existing research results, this paper finds that systematic studies on economy, society and environment are lacking in the sustainable development of resource-based cities. We believe that it is necessary to objectively construct a scientific evaluation system and use reasonable methods for statistics, evaluation and analysis to propose a more accurate coupling governance path. In particular, we used the methods of location quotient, input industry and coupling cooperation degree analysis in our manuscript to further verify the reliability of the results. In addition, this article takes Guangxi as an example to systematically sort out the current situation of mining economy, social governance and environmental maintenance, and analyzes the coupling effect between the factors on the basis of evaluating the development level of each dimension. The following understandings are mainly obtained.

Firstly, the spatial difference of resource endowment in Guangxi is large, and the non-ferrous resource reserves in some regions still have significant comparative advantages. The supporting effect of resource industry on regional economy has weakened, but the relationship between resource industry sector and other sectors is still close. Among them, the sectors of *Mining & Dressing of Nonmetal Minerals and others*, *Petroleum Processing*, *Coke Products & Processing of Nuclear Fuel* and *Smelting & Pressing of Metals* have obvious advantages. However, the consumption of energy and electricity in the resource industry has not yet reached its peak, and it will continue to increase in the future. Job security in the resources industry has declined. In addition, although the intensity of technological innovation continues to increase, it is at a relatively low level in China. The pollution emission intensity of the resource industry fluctuates, but it generally shows an upward trend, and coke, raw coal and other gases are the key factors leading to pollution in the resource industry sector. There is uncertainty in the intensity of environmental conservation investment in the resource industry, and obvious results have been achieved in pollution control.

Secondly, as for the evaluation results, the development level of mining economy and environmental conservation is relatively stable, and the development level of social governance shows an overall upward trend. The development level of mining economy in western Guangxi is higher than that in eastern Guangxi. The development level of social governance in central cities is higher than that in other regions. The distribution of environmental conservation development level is relatively random, but the resource-rich region presents a dual driven situation of resource industry and environmental conservation. As for the spatial distribution characteristics, the spatial distribution of each dimension is relatively random, showing a global positive aggregation effect only in the mining economic system, and a global negative discrete effect in the environmental conservation system. In addition, the key indicators restricting the mining economy, social governance and environmental conservation include the proportion of medium-sized or above mining areas, the proportion of secondary and tertiary industries, the number of employees and wages in the resource industry sector, carbon and industrial wastewater emissions.

Thirdly, the coupling effect between mining economy, social governance and environmental conservation is uncertain, and the coupling effect between mining economy and social governance is stronger than that between mining economy and environmental conservation. Except for Qinzhou, Laibin and Liuzhou, the coupling effect among mining economy, social governance and environmental conservation in other cities is above the level of general state. The coupling effect between mining economy and social governance in central cities is relatively strong. The coupling effect between mining economy and environmental conservation in resource-rich regions is higher than that in other regions.

### 4.2 Discussion

From the collaborative governance experience of mining economy, social governance and environmental conservation in Guangxi, it can be seen that resource and environmental policy changes lead to the uncertainty of development level and coupling effect. First of all, the material consumption of the resource industry and the external guarantee of resource products lead to the weakening of resource endowment advantages, and the resource industry lacks sustainable mineral resource supply. In addition, the constraint effect of environmental policy on resource industry leads to economic transformation, and the supporting role of resource industry on regional economy weakens. Secondly, the decoupling of mining economy, social governance and environmental conservation reflects the independence of regional economic and social governance. In the face of external policy interference, regions will formulate differentiated strategies according to their actual needs for economic and social development, resulting in the failure to form a cross-regional collaborative governance structure. Thirdly, although the advantageous regions and central cities of resource endowment have potential in their respective advantageous sectors, they are limited by the weak overall economic level of Guangxi, and their spatial radiation effect is relatively poor. Finally, according to the evaluation results and the dynamic change trend of coupling coordination degree, the resilience of mining economy, social governance and environment conservation in Guangxi is poor, which would not cope with impact of sudden events.

Therefore, under the background of China’s promotion of high-quality development, We must make efforts from the following aspects in order to realize the coupling and coordinated governance mode of mining economy, social governance and environmental conservation.

Firstly, the government should find out the latest characteristics of regional resource endowment and develop characteristic resource industries based on resource endowment conditions. The mineral resources in China are large in scale and diverse in variety, but some important mineral resources are at a disadvantage. The Chinese government is promoting a new round of breakthroughs in the exploration of strategic key minerals, which is conducive to re-sorting the current resource endowment of China. Industrial sectors need to develop characteristic industries according to the existing resource endowment conditions and rely on sustainable mineral resource supply, so as to realize the independent control of the front end of the industrial chain and enhance the economic competitiveness of China’s resource industry.

Secondly, we should focus on enhancing the technological innovation ability of the resource industry, realizing the clean transformation, promoting the development of high-end industries, and building an integrated "resource-asset-capital" industrial chain management mode. In other words, in addition to the development of advantageous resource industries, we also need to pay attention to the self-ability improvement of the resource industry from a macro perspective, including technology, management, finance and other aspects. A high-quality mining circular economy system is an important factor supporting the mining economy, social governance and environmental conservation. It is necessary to achieve high-quality supply at the resource end, and at the same time, it is necessary to use technological innovation methods to continuously explore the value of resource products, and finally use the capital market to drive the sustainable development of the mining market.

Thirdly, the spatial barriers of the circulation of resource products, technical talents and financial capital must be broken, and the development model of mining economy with cross-regional collaborative governance should be explored. Generally speaking, the development of mining economy has significant spatial differences, which leads to differences in social governance and environmental conservation in different regions. Government departments should achieve cross-regional circulation of resource products through policy guidance, and talents and funds also need to cooperate among different regions. Regional cooperation can be considered to realize the common development of resource-based cities.

Finally, Resource-based cities should rely on the policy advantages of ethnic regional autonomy and strive for better policy conditions for the development of resource industry. We are concerned that most resource-based cities have unique policy advantages, which may come from ethnic groups, industries, mountains, rivers, etc. These advantageous areas can strive for more conditions for mining economic development, such as capital, environment, talent and jurisdiction, which can reduce the impact of industrial transformation. Even, resource-based cities can encourage the development of mining economy by formulating some special policies.

## Supporting information

S1 Table Evaluation system of mining economy, social governance and environmental conservation. (DOCX)

S2 Table The advantage ratio of mineral resources reserves in various cities of Guangxi. (DOCX)

S3 Table The advantage ratio of resource industry sectors in various cities of Guangxi. (DOCX)
