# Supplementary material for: Spatio-temporal heterogeneity and coupling effect of mining economy, social governance and environmental conservation: Evidence from Guangxi Zhuang Autonomous Region, China
Source: PLoS One. 2024 Apr 16;19(4):e0301585. doi: 10.1371/journal.pone.0301585 (PMC11020948; doi:10.1371/journal.pone.0301585)
Supplement: S1 Table — (DOCX) [file pone.0301585.s001.docx]

**S1 Table. Evaluation system of mining economy, social governance and environmental conservation**

| **Target** | **Criterion** | **Element** | **Selection basis** | **Stats** |
| --- | --- | --- | --- | --- |
| Mining economy | Resource guarantee ability | **X_1_.** The proportion of medium and above mineral deposits | Exploitation potential of mineral resources | positive |
|  |  | **X_2_.** The growth rate of energy reserves | Energy extraction and security capabilities | positive |
|  |  | **X_3_.** The growth rate of major mineral reserves | Mineral resources exploitation and security capabilities | positive |
|  | Regional trade support | **X_4_.** The profit contribution rate of mining sectors | The extent to which the mining sector contributes to the regional economy | positive |
|  |  | **X_5_.** The fixed assets investment of the mining sectors | Input of production factors in the mining sectors | positive |
|  |  | **X_6_.** The foreign trade volume of mineral resource products | Mineral resources trade circulation activity | positive |
|  | Industry correlation effect | **X_7_.** The driving role of the mining sectors | The material supply from the mining sector to other industrial sectors | positive |
|  |  | **X_8_.** The pulling role of the mining sectors | The material demands of the mining sector on other industrial sectors | positive |
|  | Effect of industrial transformation | **X_9_.** The contribution of industry to GDP | It partly reflects the contribution of the mining sector to GDP and the degree of transformation | positive |
|  |  | **X_10_.** The proportion of secondary and tertiary industrial structure | The transformation rate of the overall industrial structure in the region | positive |
| Social governance | Employment security | **X_11_.** Unemployment rate | The overall unemployment in the labor force | negative |
|  |  | **X_12_.** The number of employees in the mining sector | The employment security of the labor force in the mining sectors | positive |
|  |  | **X_13_.** The average wages in the mining sector | The wage security of labor in mining sectors | positive |
|  | Resource appropriation | **X_14_.** Energy consumption in the mining sectors | The energy consumption intensity of mining production | negative |
|  |  | **X_15_.** Electricity consumption in the mining sectors | The electricity consumption intensity of mining production | negative |
|  | Technological innovation | **X_16_.** The proportion of R&D expenditure in mining sector | The investment of technological innovation funds in mining sectors | positive |
| Environmental conservation | Pollution discharge | **X_17_.** The proportion of carbon emissions from the mining sectors | The extent of carbon pollution from mining production | negative |
|  |  | **X_18_.** Wastewater discharge from the mining sector | The discharge of wastewater from mining production | negative |
|  |  | **X_19_.** Emissions from the mining sector | The discharge of industrial emissions from mining production | negative |
|  |  | **X_20_.** Solid waste emissions from the mining sector | The discharge of industrial solid waste from mining production | negative |
|  | Pollution control | **X_21_.** Investment in industrial pollution control | It partly reflects the investment in mining pollution control | negative |
|  | Governance effectiveness | **X_22_.** Geological hazard number | The occurrence of disasters in the mining field | negative |
|  |  | **X_23_.** The proportion of days with good air | It partly reflects the effectiveness of environmental pollution control | positive |
