# Supplementary material for: Spatio-temporal heterogeneity and coupling effect of mining economy, social governance and environmental conservation: Evidence from Guangxi Zhuang Autonomous Region, China
Source: PLoS One. 2024 Apr 16;19(4):e0301585. doi: 10.1371/journal.pone.0301585 (PMC11020948; doi:10.1371/journal.pone.0301585)
Supplement: S2 Table — (DOCX) [file pone.0301585.s002.docx]

**S2 Table. The advantage ratio of mineral resources reserves in various cities of Guangxi**

| **Resources** | **Nanning** | **Guilin** | **Liuzhou** | **Hechi** | **Baise** | **Chongzuo** | **Hezhou** | **Wuzhou** | **Yulin** | **Laibin** | **Guigang** | **Qinzhou** | **Fangchenggang** | **Beihai** |
| --- | --- | --- | --- | --- | --- | --- | --- | --- | --- | --- | --- | --- | --- | --- |
| Coal | -0.12 | -0.97 | -0.98 | 2.01 | 2.58 | 1.75 | -0.71 | -1.00 | -1.00 | 9.77 | -1.00 | -0.83 | -1.00 | -0.62 |
| Petroleum | -1.00 | -1.00 | -1.00 | -1.00 | 15.92 | -1.00 | -1.00 | -1.00 | -1.00 | -1.00 | -1.00 | -1.00 | -1.00 | -1.00 |
| Iron | 0.07 | 0.98 | -0.38 | -0.26 | -0.93 | 4.59 | 3.77 | -0.88 | 0.03 | -0.96 | -0.97 | -0.96 | -0.96 | -0.99 |
| Manganese | -0.94 | -0.58 | -0.79 | -0.29 | 5.45 | 10.98 | -0.76 | -0.95 | -0.97 | 0.11 | -0.53 | -1.00 | -0.97 | -1.00 |
| Vanadium | 2.13 | 1.73 | -1.00 | -0.82 | -1.00 | 0.70 | -1.00 | -1.00 | -1.00 | -1.00 | -1.00 | -1.00 | -1.00 | -1.00 |
| Bauxite | -0.83 | -1.00 | -1.00 | -0.88 | 10.93 | 4.53 | -1.00 | -1.00 | -1.00 | 0.84 | -1.00 | -1.00 | -1.00 | -1.00 |
| Lead | -0.71 | 1.91 | -0.96 | -0.10 | -1.00 | 0.08 | -0.59 | 3.61 | -0.93 | 1.40 | 3.44 | -1.00 | -1.00 | -1.00 |
| Zinc | -0.92 | -0.32 | -0.50 | 8.99 | -0.92 | 0.65 | -0.69 | 0.08 | -0.86 | 6.99 | -0.18 | -1.00 | -1.00 | -1.00 |
| Antimony | -0.23 | -0.78 | -0.93 | 9.88 | 3.95 | -1.00 | -0.51 | -1.00 | -0.45 | -1.00 | -1.00 | -1.00 | -0.92 | -1.00 |
| Copper | 0.22 | 0.11 | -0.03 | -0.66 | 2.63 | -1.00 | -0.36 | -1.00 | -0.87 | 5.33 | -0.40 | -1.00 | -1.00 | -1.00 |
| Tungsten | 0.60 | -0.11 | -1.00 | -0.45 | -1.00 | -0.20 | 3.04 | 1.38 | 2.51 | -1.00 | -1.00 | -1.00 | -1.00 | -1.00 |
| Tin | -1.00 | -0.70 | -0.71 | 18.96 | -0.99 | -1.00 | 1.90 | -1.00 | -0.98 | -1.00 | -0.92 | -1.00 | -0.94 | -1.00 |
| Cobalt | -1.00 | -1.00 | -1.00 | -1.00 | -1.00 | 26.98 | -1.00 | -1.00 | -1.00 | -1.00 | -1.00 | -1.00 | -1.00 | -1.00 |
| Titanium | -1.00 | -1.00 | -1.00 | 5.32 | 0.74 | -1.00 | -0.98 | 10.99 | -0.94 | -1.00 | -1.00 | -0.97 | -1.00 | -1.00 |
| Nickel | -1.00 | -1.00 | 1.25 | 15.22 | -1.00 | -1.00 | -1.00 | -1.00 | -1.00 | -1.00 | -1.00 | -1.00 | -1.00 | -0.62 |
| Molybdenum | -1.00 | -1.00 | -1.00 | -1.00 | -1.00 | -1.00 | 0.07 | 5.04 | 7.66 | -1.00 | -1.00 | -1.00 | -1.00 | -1.00 |
| Gold | -0.73 | -0.38 | -0.97 | 0.41 | 8.01 | -0.22 | 2.55 | -0.10 | -0.89 | -1.00 | 0.52 | -1.00 | -0.99 | -0.99 |
| Silver | 0.46 | -1.00 | -1.00 | -0.17 | -1.00 | -1.00 | 2.57 | 0.46 | 1.51 | 6.25 | -0.15 | -1.00 | -1.00 | -1.00 |
| Kaolin | -0.93 | -0.98 | -0.98 | -1.00 | -1.00 | -1.00 | -1.00 | -0.94 | -0.43 | -1.00 | -1.00 | -0.99 | -1.00 | -1.00 |
| Granite | -0.92 | -0.20 | -1.00 | -1.00 | -1.00 | -1.00 | 16.41 | 2.79 | -0.35 | -1.00 | -0.95 | -0.09 | 0.28 | -1.00 |
| Talc | -0.66 | 8.17 | -1.00 | -0.58 | -0.95 | -1.00 | -1.00 | -1.00 | -1.00 | -1.00 | -1.00 | -1.00 | -1.00 | -1.00 |
| Phosphate | -0.99 | -1.00 | -0.93 | -1.00 | 5.69 | 7.20 | 0.06 | -0.50 | 2.05 | -1.00 | -1.00 | -1.00 | -1.00 | -1.00 |
| Pyrite | -0.95 | -0.92 | -0.98 | 20.13 | 0.56 | -1.00 | -0.96 | -0.92 | -0.92 | -1.00 | -0.98 | -1.00 | -1.00 | -1.00 |
| Mirabilite | 3.72 | -1.00 | -1.00 | -1.00 | -1.00 | -1.00 | -1.00 | -1.00 | -1.00 | -1.00 | -1.00 | -1.00 | -1.00 | -1.00 |
| Clay | 1.57 | -1.00 | -1.00 | -1.00 | -1.00 | -1.00 | -1.00 | -1.00 | -1.00 | 9.12 | -1.00 | -1.00 | -1.00 | -1.00 |
| Bentonite | -0.99 | -1.00 | -1.00 | -0.89 | 0.15 | 24.27 | -1.00 | -0.95 | -1.00 | -1.00 | -1.00 | -1.00 | -0.78 | -1.00 |
| Gypsum | -1.00 | -1.00 | -1.00 | -1.00 | -1.00 | -1.00 | -1.00 | -1.00 | -1.00 | -0.74 | -1.00 | 11.00 | -1.00 | -1.00 |
| Limestone | -0.42 | 0.15 | -0.36 | 0.20 | 1.20 | 1.88 | -0.08 | -0.79 | 0.22 | 0.51 | 1.80 | -0.98 | -0.41 | -1.00 |
| Fluorite | -1.00 | 2.60 | -1.00 | -1.00 | -1.00 | -1.00 | 0.23 | -0.34 | 5.30 | -1.00 | -1.00 | -1.00 | 1.27 | -0.62 |
| Wollastonite | -1.00 | 0.92 | -1.00 | -1.00 | -1.00 | -1.00 | 23.63 | -1.00 | -1.00 | -1.00 | -1.00 | -1.00 | -1.00 | -1.00 |
| Marble | -1.00 | -0.53 | -1.00 | -1.00 | -1.00 | 0.27 | 26.59 | -1.00 | -1.00 | -1.00 | -1.00 | -1.00 | -1.00 | -0.99 |
| Barite | -0.97 | -0.45 | 0.74 | -0.46 | -0.21 | 0.04 | -1.00 | -1.00 | -1.00 | 17.77 | -1.00 | -1.00 | -1.00 | -1.00 |
