# Supplementary material for: Spatio-temporal heterogeneity and coupling effect of mining economy, social governance and environmental conservation: Evidence from Guangxi Zhuang Autonomous Region, China
Source: PLoS One. 2024 Apr 16;19(4):e0301585. doi: 10.1371/journal.pone.0301585 (PMC11020948; doi:10.1371/journal.pone.0301585)
Supplement: S3 Table — (DOCX) [file pone.0301585.s003.docx]

**S3 Table. The advantage ratio of resource industry sectors in various cities of Guangxi**

| **Sectors** | **Nanning** | **Guilin** | **Liuzhou** | **Hechi** | **Baise** | **Chongzuo** | **Hezhou** | **Wuzhou** | **Yulin** | **Laibin** | **Guigang** | **Qinzhou** | **Fangchenggang** | **Beihai** |
| --- | --- | --- | --- | --- | --- | --- | --- | --- | --- | --- | --- | --- | --- | --- |
| **A_1_** | -1.00 | -1.00 | -1.00 | 3.42 | 13.58 | 0.34 | -1.00 | -1.00 | -1.00 | -1.00 | -1.00 | -1.00 | 0.03 | -1.00 |
| **A_2_** | -1.00 | -1.00 | -1.00 | -1.00 | -1.00 | -1.00 | -1.00 | -1.00 | -1.00 | -1.00 | -1.00 | -1.00 | -1.00 | 16.15 |
| **A_3_** | -1.03 | -1.03 | -0.95 | 9.28 | -1.55 | 3.36 | -0.50 | 6.16 | -0.99 | -0.41 | -0.90 | -0.51 | 0.12 | -1.00 |
| **A_4_** | -0.67 | 0.62 | -0.97 | -1.06 | 0.65 | -1.00 | 1.39 | 3.43 | -0.87 | -0.20 | 0.25 | -1.00 | 1.12 | -0.15 |
| **A_5_** | -1.00 | -1.00 | -0.99 | -1.00 | -1.19 | -1.00 | -1.00 | -1.00 | -1.00 | -1.00 | -1.00 | 6.27 | -1.00 | 9.31 |
| **A_6_** | -0.24 | -0.34 | -0.71 | -0.60 | -0.20 | 1.68 | 0.29 | 0.58 | 0.46 | -0.04 | 2.50 | -0.83 | 0.04 | -0.64 |
| **A_7_** | -0.97 | -0.97 | 2.24 | -1.39 | 1.66 | -0.57 | -0.96 | 0.64 | -0.94 | -1.79 | -0.48 | -0.82 | 5.64 | -0.87 |
| **A_8_** | 1.43 | -0.79 | -0.21 | -1.00 | -0.91 | -0.91 | -1.49 | -0.45 | 0.52 | -0.97 | -0.94 | -0.80 | -0.82 | -0.70 |
